# Comparative Outcomes of Surgical and Non-Surgical Treatment in Patients with Lumbar Radiculopathy: A Six-Month Analysis of Quality of Life and Functional Status

**DOI:** 10.3390/jcm15218462

**Published:** 2026-10-30

**Authors:** Elvis Mahmutović, Teodora Safiye, Slaviša Minić, Vesna Zeljković, Zoran Bajin, Zerina Salihagić, Aldina Ajdinović, Hristina Komatina, Medina Gusinac Bošnjak, Almasa Sokolović, Zana Dolićanin, Emir Biševac

**Affiliations:** 1 Department of Medical Sciences, State University of Novi Pazar, 36300 Novi Pazar, Serbia; ehmahmutovic@np.ac.rs (E.M.); sminic@np.ac.rs (S.M.); vzeljkovic@np.ac.rs (V.Z.); zbajin@np.ac.rs (Z.B.); zsalihagic@np.ac.rs (Z.S.); aajdinovic@np.ac.rs (A.A.); hkomatina@np.ac.rs (H.K.); mgusinacbosnjak@np.ac.rs (M.G.B.); asokolovic@np.ac.rs (A.S.); zdolicanin@np.ac.rs (Z.D.); ebisevac@np.ac.rs (E.B.); 2 Department of Psychology, State University of Novi Pazar, 36300 Novi Pazar, Serbia

**Keywords:** radiculopathy, diskectomy, rehabilitation, quality of life, recovery of function

## Abstract

**Background/Objectives**: Lumbar radiculopathy can be managed non-surgically or surgically; however, comparative changes in quality of life and functional status between these treatment pathways remain clinically relevant. Therefore, this study aimed to compare six-month outcomes in patients treated non-surgically and those treated with lumbar microdiscectomy followed by rehabilitation. **Methods**: A prospective, non-randomized comparative cohort study included 100 patients with discogenic lumbar radiculopathy: 50 treated non-surgically and 50 treated by lumbar microdiscectomy. Both groups underwent medical rehabilitation and were assessed at admission, three months, and six months using the Short Form-36 Health Survey (SF-36), including the Physical Component Summary (PCS) and Mental Component Summary (MCS), and the Oswestry Disability Index (ODI). **Results**: PCS improved from 35.5 to 50.8 in the non-surgical group and from 28.8 to 49.2 in the surgical group. PCS was significantly higher in non-surgically treated patients at admission (*p* < 0.001) and three months (*p* < 0.05), whereas the difference was not significant at six months (*p* = 0.064). No between-group differences were found for MCS at admission, three months, or six months (*p* = 0.104, *p* = 0.871, and *p* = 0.323, respectively). ODI decreased from 51.5% to 22.5% in the non-surgical group and from 56.1% to 23.7% in the surgical group, without significant between-group differences at any assessment (*p* = 0.109, *p* = 0.162, and *p* = 0.427). **Conclusions**: Both treatment pathways were associated with substantial improvement. The initial between-group difference in physical quality of life diminished during follow-up, while mental quality of life and disability outcomes were similar between groups.

## 1. Introduction

Lumbar radiculopathy is a clinical syndrome characterized by radiating lower-extremity pain, sensory symptoms, and, in some patients, motor or reflex impairment caused by irritation or compression of a lumbar nerve root. Lumbar disc herniation is among its most frequent structural causes and affects adults during economically and socially active years. Its burden extends beyond pain to impaired mobility, reduced participation in work and family roles, and diminished health-related quality of life [1,2].

Treatment selection is influenced by symptom severity and duration, neurological findings, imaging, patient preferences, previous treatment, and the presence of urgent surgical indications. In the absence of progressive motor deficit or cauda equina syndrome, contemporary recommendations support an initial non-surgical approach. This may combine education, activity modification, exercise, physical therapy, medication, and selected interventional procedures [3,4,5]. Surgery, most commonly discectomy or microdiscectomy, is considered when disabling radicular symptoms persist despite adequate conservative management or when neurological compromise warrants decompression [6].

The principal advantage of discectomy is usually faster relief of leg pain. A recent meta-analysis of randomized trials found that surgery produced greater short-term improvement than non-surgical care, but the advantage declined over time and was small or negligible at longer follow-up [7]. Randomized-trial follow-up in chronic sciatica nevertheless indicates that appropriately selected patients may retain clinically relevant benefits from microdiscectomy [8]. Other studies comparing microdiscectomy with minimally invasive intradiscal procedures have also demonstrated substantial improvement in both treatment arms [9]. These findings make the trajectory of recovery, rather than a single endpoint, important for shared decision-making.

Non-surgical rehabilitation can reduce disability and improve quality of life in lumbar radiculopathy. A randomized trial showed meaningful improvements after routine physical therapy, with variable additional benefit from non-surgical decompression [10]. Epidural approaches may also reduce pain and disability in selected patients, although treatment effects and certainty vary across interventions [11,12]. After surgery, active rehabilitation is intended to restore mobility, confidence, strength, and participation. Recent systematic reviews support postsurgical exercise and rehabilitation, while also emphasizing heterogeneity in program content and timing [13,14,15,16].

Patient-reported outcome measures are central to evaluating these pathways. The Short Form-36 Health Survey (SF-36) captures broad physical and mental dimensions of health through its Physical Component Summary (PCS) and Mental Component Summary (MCS), whereas the Oswestry Disability Index (ODI) quantifies limitations specifically associated with low-back and radicular symptoms [17,18]. Used together, they distinguish general quality of life from condition-specific functional disability.

The two cohorts examined in the present study were previously reported separately: one publication described non-surgically treated patients [3], and another reported outcomes following lumbar microdiscectomy [4]. Those reports demonstrated improvement within each treatment pathway but did not directly evaluate between-group differences in the longitudinal course of patient-reported quality of life and functional disability. The present study therefore extends the previously published cohort findings by providing a new direct comparative analysis of the original combined prospective cohort, with PCS, MCS, and ODI compared at matched assessment points over six months.

Although previous studies have compared surgical and non-surgical management of lumbar radiculopathy, the course of functional recovery and changes in health-related quality of life during subsequent rehabilitation remain clinically relevant outcomes. By evaluating both cohorts within the same specialized rehabilitation setting at baseline, three months, and six months, the study sought to describe their respective recovery trajectories rather than establish the comparative causal effectiveness of the initial treatment strategies.

The aim of this exploratory prospective study was to compare health-related quality of life and functional disability between surgically and non-surgically treated patients with lumbar radiculopathy at rehabilitation admission and at three- and six-month follow-up assessments, as well as to examine changes in these outcomes separately within each group. No single primary outcome or formal directional hypothesis was prespecified.

## 2. Materials and Methods

### 2.1. Study Design, Participant Selection, and Treatment Protocols

This was a prospective, comparative cohort study conducted at the Special Hospital for Progressive Muscular and Neuromuscular Diseases in Novi Pazar, Serbia.

The study included 100 patients with discogenic lumbar radiculopathy. Fifty had been managed non-surgically, and 50 had undergone lumbar microdiscectomy before entering the rehabilitation program. Participants were randomly sampled within two pre-existing treatment strata using random-number procedures. Treatment allocation was determined independently of the research protocol. Patients in the surgical cohort had previously undergone lumbar microdiscectomy at other healthcare institutions based on established clinical indications for surgical intervention and were subsequently referred to the participating tertiary healthcare institution for postoperative rehabilitation. Patients in the non-surgical cohort were referred to the same institution for conservative rehabilitation without a previously established indication for surgery. The investigators were not involved in surgical decision-making or treatment allocation. Accordingly, the study prospectively compared rehabilitation outcomes between two pre-existing, clinically determined treatment cohorts, without random allocation to surgical or non-surgical treatment.

Patients were referred to the participating tertiary rehabilitation institution from primary and secondary healthcare facilities by neurologists or specialists in physical and rehabilitation medicine, while patients who had undergone lumbar microdiscectomy were referred for postoperative rehabilitation by neurosurgeons. The diagnosis had been established before referral on the basis of neurological examination and magnetic resonance imaging (MRI). All patients had an MRI report interpreted by a radiologist. Thus, the initial diagnostic evaluations were performed independently of the research protocol.

Detailed information on the specific clinical factors underlying the original treatment decisions, including the extent and duration of previous conservative treatment, neurological findings at the time of treatment decision, and the role of patient preference, was not systematically recorded in the study dataset and could not be reliably reconstructed retrospectively. The original study was not prospectively registered in a publicly accessible study or clinical trial registry.

Participant recruitment was conducted from 1 October 2014 to 1 March 2016, following Ethics Committee approval obtained on 17 September 2014. Among patients meeting the eligibility criteria and entering the random selection process, 212 were in the non-surgical treatment stratum and 84 in the surgical treatment stratum. Using random-number procedures, 50 participants were selected from each stratum, resulting in a final study sample of 100 participants. All 100 enrolled participants completed the assessments at the start of rehabilitation, 3 months, and 6 months, and no participant withdrew during follow-up.

Eligible participants were men and women aged 20–65 years with a confirmed diagnosis of discogenic lumbar pain syndrome with lumbar radiculopathy or lumbar disc herniation. Participants had to be oriented to time, place, and person; able to provide written informed consent; and able to follow the prescribed treatment and assessment protocol. Exclusion criteria included pregnancy, participation in another clinical study, and inability to comply with the study protocol.

In addition to severe cardiovascular disorders and active malignant disease, other comorbidities and the presence of medical implants were considered when determining the suitability of individual rehabilitation interventions. Certain conditions or implants could contraindicate specific physical therapy modalities or necessitate their modification or omission. Eligibility for the rehabilitation program and the appropriateness of individual interventions were assessed by a specialist in physical and rehabilitation medicine based on clinical examination and available medical documentation. When clinically indicated, the rehabilitation program was adapted to the patient’s individual circumstances.

Both treatment cohorts underwent a two-week rehabilitation program according to the institution’s standard therapeutic protocol. Rehabilitation constituted the primary therapeutic approach during the study period. The program included ergonomic education and a structured exercise regimen consisting of Regan exercises, McKenzie exercises, and lumbo–abdominal stabilization exercises. Physical therapy modalities included interferential current therapy (1–100 Hz) applied through vacuum electrodes for 20 min, magnetotherapy at a magnetic flux density of 20 mT for 30 min, and therapeutic ultrasound at an intensity of 0.8 W/cm^2^ for 5 min, administered before kinesitherapy.

Pharmacological management followed the same institutional analgesic treatment protocol in both cohorts. When clinically indicated, analgesic medications were used for short-term symptom relief to facilitate participation in the rehabilitation program. The selection of specific medications was individualized according to each patient’s clinical condition and therapeutic needs. Detailed patient-level information on medication types, dosages, and exact treatment duration was not available for the present analysis.

Upon discharge, all patients were instructed to continue performing the prescribed exercises independently and to adhere to ergonomic recommendations in their home environment. The admission assessment corresponded to admission/start of rehabilitation in the non-surgical cohort and to the beginning of rehabilitation immediately after surgery in the surgical cohort.

### 2.2. Outcome Measures and Assessment Procedures

Outcome data were collected exclusively by members of the research team who were not involved in treatment or rehabilitation delivery. At baseline, participants generally completed the SF-36 and ODI questionnaires independently after receiving standardized instructions, with interviewer assistance provided when necessary. At three- and six-month follow-up, data were collected predominantly through structured telephone interviews, with a smaller number of responses obtained through face-to-face interviews, email, or postal questionnaires. The researchers administered the questionnaires and recorded patients’ responses without independently rating their functional status or health-related quality of life. Although the researchers were aware of participants’ treatment-group allocation, they were not involved in treatment decisions or therapeutic interventions.

Participants were assessed at three predefined time points: at the start of rehabilitation (baseline), three months later, and six months later, allowing changes in health-related quality of life and functional status to be evaluated longitudinally.

#### 2.2.1. Short Form-36 Health Survey (SF-36v2)

Health-related quality of life was assessed using the officially provided Serbian-language version of the Short Form-36 Health Survey, version 2 (SF-36v2), obtained under license (License No. QM024915) and administered without modification. All participants were proficient in Serbian. The questionnaire comprises eight health domains: Physical Functioning (PF), Role Physical (RP), Bodily Pain (BP), General Health (GH), Vitality (VT), Social Functioning (SF), Role Emotional (RE), and Mental Health (MH).

Responses to individual items were entered into Health Outcomes Scoring Software, version 4.5 (QualityMetric Incorporated, Lincoln, RI, USA), which automatically calculated the eight domain scores and the Physical Component Summary (PCS) and Mental Component Summary (MCS) scores using its built-in scoring algorithms. The eight domain scores were expressed on a 0–100 scale, whereas PCS and MCS were calculated separately as norm-based component summary measures and were not restricted to the same numerical range. Higher scores indicate better health-related quality of life.

#### 2.2.2. Oswestry Disability Index (ODI)

Functional disability was assessed using an existing Serbian-language version of the Oswestry Disability Index (ODI), reproduced in the rehabilitation textbook Klinička kineziterapija by Jevtić [17]. The questionnaire was administered without translation or modification by the research team [17].

The administered version comprised ten sections: Pain Intensity, Personal Care, Lifting, Walking, Sitting, Standing, Sleeping, Work/Housework, Social Life, and Traveling. Unlike the standard ODI, this version includes a “Work/Housework” section instead of “Sex Life.” This alternative ODI structure has been psychometrically evaluated by Davidson (2008), who reported adequate fit to the Rasch measurement model [18].

Each section contains six response options scored from 0 to 5. The total ODI score was calculated by dividing the sum of the item scores by the maximum possible score and multiplying by 100, yielding a percentage score ranging from 0% to 100%. Higher scores indicate greater functional disability, in contrast to the interpretation of SF-36 scores.

### 2.3. Statistical Analysis of Repeated Measurements

Statistical processing was performed with IBM SPSS Statistics version 20.0 (IBM Corp., Armonk, NY, USA) and Health Outcomes Scoring Software, version 4.5 (QualityMetric Incorporated, Lincoln, RI, USA). Continuous data were summarized using means and standard deviations when available, while categorical data were presented as counts and percentages. Admission categorical characteristics were compared with the chi-square test or Fisher’s exact test, and continuous variables with Student’s t test or the Mann–Whitney U test according to distribution. Change in numerical outcomes across the three assessments within each cohort was tested using repeated-measures analysis of variance in the General Linear Model framework, with Greenhouse-Geisser correction where appropriate. Between-group outcome values at each assessment were compared with the Mann–Whitney U test. Statistical significance was defined as *p* < 0.05. Absolute admission-to-six-month changes were calculated descriptively. The source analysis did not include a formal treatment-by-time interaction or an adjusted longitudinal treatment effect.

#### Sample Size Considerations

Before recruitment, sample size estimation was performed using the online Sample Size Calculator (Calculator.net), assuming a 95% confidence level and an 8% margin of error. This calculation was used to guide sample selection and was not based on a prespecified primary outcome, expected effect size, or target statistical power. Therefore, no formal outcome-specific a priori power analysis was performed. The final sample comprised 100 participants, with 50 in each treatment group.

### 2.4. Ethical Considerations

The study protocol and written informed consent materials received approval from the Ethics Committee of the Special Hospital for Progressive Muscular and Neuromuscular Diseases, Novi Pazar, Serbia (protocol code 1048; date of approval: 17 September 2014). The study was conducted in accordance with the Declaration of Helsinki and principles of good clinical practice. All participants provided written informed consent before inclusion.

## 3. Results

### 3.1. Participant Characteristics

All 100 participants completed assessments at admission, three months, and six months. Mean age was 48.20 ± 6.49 years in the non-surgical cohort and 47.12 ± 7.63 years in the surgical cohort (*p* = 0.448). Sex distribution did not differ significantly between groups (*p* = 0.405). Sudden onset was more frequent in the non-surgical group, whereas gradual onset predominated in the surgical group (*p* < 0.05). Previous lumbar episodes were similarly distributed (*p* = 0.659) (Table 1).

### 3.2. SF-36 Physical Component Summary

PCS increased significantly across the three assessments in both groups (non-surgical: F = 450.221, *p* < 0.001; surgical: F = 490.721, *p* < 0.001). The non-surgical group had a higher PCS at admission (35.5 vs. 28.8; *p* < 0.001) and at three months (44.7 vs. 42.8; *p* < 0.05). At six months, PCS values were numerically close and the between-group difference was not statistically significant (50.8 vs. 49.2; *p* = 0.064). The descriptive admission-to-six-month increase was 15.3 points in the non-surgical group and 20.4 points in the surgical group (Table 2).

### 3.3. SF-36 Mental Component Summary

MCS improved significantly over time in the non-surgical (F = 106.543, *p* < 0.001) and surgical (F = 72.055, *p* < 0.001) groups. No significant between-group difference was observed at admission (40.6 vs. 37.8; *p* = 0.104), three months (44.8 vs. 45.2; *p* = 0.871), or six months (52.6 vs. 52.5; *p* = 0.323). The descriptive six-month gains were 12.0 and 14.7 points, respectively.

### 3.4. Oswestry Disability Index

ODI decreased significantly across follow-up in both cohorts (non-surgical: F = 432.810, *p* < 0.001; surgical: F = 1341.180, *p* < 0.001). Between-group differences were not statistically significant at admission (51.5% vs. 56.1%; *p* = 0.109), three months (36.0% vs. 38.9%; *p* = 0.162), or six months (22.5% vs. 23.7%; *p* = 0.427). The absolute six-month reduction was 29.0 percentage points in the non-surgical group and 32.4 percentage points in the surgical group. Overall, within-group changes were statistically significant for all three outcomes in both cohorts, whereas significant between-group differences were observed only for SF-36 PCS at admission and at three months; the complete statistical comparisons are summarized in Table 3.

In addition to the PCS and MCS scores, the results for all eight SF-36v2 domains (Physical Functioning, Role Physical, Bodily Pain, General Health, Vitality, Social Functioning, Role Emotional, and Mental Health) are presented in Supplementary Table S2 as mean ± standard deviation (SD) for both treatment groups at rehabilitation admission and at three- and six-month follow-up assessments. Improvements in individual domain scores were observed in both groups during follow-up.

**Figure 1:**
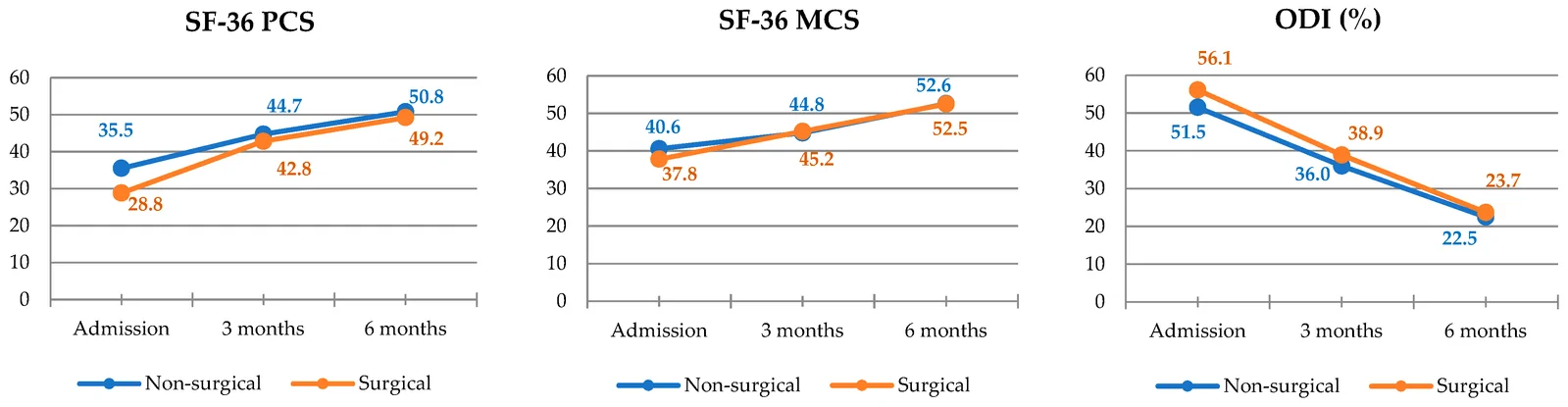
Mean SF-36 PCS, SF-36 MCS, and ODI values at admission, three months, and six months. Values above each panel are between-group *p*-values in chronological order. Higher PCS/MCS and lower ODI indicate better outcomes.

To further assess the clinical relevance of the observed changes in functional disability, an exploratory responder analysis was performed using a reduction of at least 10 percentage points in ODI as a threshold for clinically meaningful improvement. At six months, 49 of 50 patients (98.0%) in the non-surgical group and all 50 patients (100.0%) in the surgical group achieved this threshold. The mean SF-36 PCS, SF-36 MCS, and ODI values at admission, three months, and six months are presented in Figure 1.

During the six-month follow-up period, no additional surgical procedures or complications were reported in either treatment group. Information regarding these events was obtained through patient follow-up. Symptom recurrence and hospital readmissions were not specifically assessed.

## 4. Discussion

This prospective comparative cohort study provides a longitudinal comparison of patient-reported quality of life and functional disability between patients managed non-surgically and those treated with lumbar microdiscectomy followed by medical rehabilitation. The principal finding was that both treatment pathways were accompanied by substantial improvement in physical and mental health-related quality of life and in condition-specific disability during six months of follow-up. The surgical cohort entered rehabilitation with a significantly lower PCS than the non-surgical cohort. This difference remained statistically significant at three months but was no longer significant at six months. In contrast, MCS and ODI did not differ significantly between groups at any assessment. The data therefore show convergence in the principal patient-reported outcomes rather than evidence that one strategy was uniformly superior across all domains and time points.

The physical component followed the most distinctive between-group pattern. PCS increased by 15.3 points in the non-surgical cohort and 20.4 points in the surgical cohort. The larger numerical gain after surgery should not be interpreted as a statistically proven superiority of change because the original analysis compared groups at each assessment and did not test a treatment-by-time interaction. Moreover, the surgical group began from a lower PCS, creating more room for improvement. Early postsurgical restrictions, fear of movement, and the immediate effects of surgery may also have contributed to the lower value recorded at the start of rehabilitation.

The disappearance of the PCS difference is consistent with contemporary comparative evidence. The 2023 BMJ meta-analysis reported that discectomy offers faster relief of sciatica, but the magnitude of its advantage over non-surgical care diminishes with time [7]. In chronic sciatica, Bailey and colleagues found better two-year outcomes after microdiscectomy than after standardized non-surgical care, while their timing analysis suggested that delaying surgery after prolonged symptoms can compromise recovery [8,19]. Large observational studies also associate longer symptom duration with poorer postsurgical outcomes [20,21]. These findings support timely surgical referral for selected patients without implying that all patients require surgery.

MCS improved continuously in both cohorts and was nearly identical at six months. Mental health may recover as pain, uncertainty, sleep disturbance, dependence, and limitations in social participation diminish. The absence of a between-group difference suggests that the route to symptom control may be less important for mental quality of life than successful recovery itself. It also supports combining physical rehabilitation with education, reassurance, and attention to psychosocial barriers regardless of treatment pathway.

ODI declined from the range indicating severe disability at admission to the range indicating moderate disability at six months in both groups. The six-month values differed by only 1.2 percentage points. Contemporary trials and systematic reviews similarly show that both surgical and non-surgical interventions can yield meaningful functional improvement, even when surgery provides earlier pain relief [7,8,9,10,11]. The present results add a pragmatic perspective: when both pathways are followed by structured rehabilitation, condition-specific disability may become closely aligned over a six-month horizon.

Rehabilitation is a shared element that may partly explain this convergence. Evidence for postsurgical programs is heterogeneous, but recent reviews generally support active exercise for reducing pain and disability and improving quality of life after lumbar disc surgery [13,14,15]. Exercise is also a core component of non-surgical care [10,16]. In the present study, both groups received the institution’s rehabilitation protocol and ergonomic education, providing a common framework for progressive functional restoration.

Treatment selection should therefore remain individualized. Current WFNS recommendations support conservative management as the first-line strategy for most patients without urgent neurological indications and support surgery for persistent disabling symptoms or neurological deterioration [5,6]. Cost-effectiveness analyses favor microdiscectomy in some patients with prolonged sciatica [22], whereas randomized studies of intradiscal therapies illustrate that less invasive alternatives may be effective for selected candidates [9,23]. Recurrence risk and patient-specific prognostic factors should also be incorporated into counselling [24,25].

The improvements observed in SF-36v2 and ODI scores over the six-month follow-up period indicate substantial positive changes in health-related quality of life and functional status in both treatment groups. The individual SF-36v2 domain scores further support these findings, particularly regarding physical functioning, bodily pain, and limitations in daily activities. The clinical relevance of the observed changes was additionally supported by an exploratory ODI responder analysis. Using a reduction of at least 10 percentage points as the threshold for clinically meaningful improvement, 98.0% of non-surgically treated patients and 100.0% of surgically treated patients met this criterion at six months. This threshold is consistent with the recommendations of Ostelo et al. regarding the interpretation of changes in functional status among patients with low back pain [26]. Although the clinical importance of individual changes may vary according to baseline status and patient characteristics, these findings suggest that the observed improvements were not limited to changes in group mean scores but were evident in nearly all participants. Nevertheless, the exploratory nature of the responder analysis and the absence of randomized treatment allocation should be considered when interpreting the findings [26].

The study has several strengths. Both cohorts were recruited in the same institution, followed over identical assessment intervals, evaluated with the same validated instruments, and exposed to a common rehabilitation framework. Complete six-month follow-up minimized attrition bias. Direct comparison of PCS, MCS, and ODI provides a broader account of recovery than pain intensity alone.

Several limitations should be considered when interpreting these findings. The non-randomized design introduces potential confounding by indication, as treatment selection may have been influenced by clinical severity and other patient characteristics. Furthermore, the baseline assessment in the surgical group was performed after surgery, whereas the non-surgical group was assessed at the beginning of rehabilitation, limiting the comparability of baseline measurements. The limited characterization of baseline clinical factors, the use of different questionnaire administration modes, and the lack of systematic information on additional treatments may have influenced the observed outcomes. The relatively small sample size and baseline differences also introduce uncertainty into between-group comparisons. Moreover, the six-month follow-up does not establish the long-term durability of improvement. Finally, because both groups received rehabilitation and no comparison group without rehabilitation was included, the independent contribution of rehabilitation to the observed improvements cannot be determined.

## 5. Conclusions

Both surgically and non-surgically treated patients with lumbar radiculopathy demonstrated improvements in health-related quality of life and functional disability during the six-month follow-up period. These findings describe favorable short-term outcomes within treatment pathways that included rehabilitation. However, given the non-randomized study design, baseline differences, and the absence of a comparison group without rehabilitation, the independent effects of rehabilitation and the comparative effectiveness of the two treatment approaches cannot be established.

## Figures and Tables

**Table 1:** Admission characteristics of the study groups.

**Characteristic**	**Non-Surgical (n = 50)**	**Surgical (n = 50)**	***p*** **-Value**
Age, years, mean ± SD	48.20 ± 6.49	47.12 ± 7.63	0.448
Female sex, n (%)	30 (60)	34 (68)	0.405
Male sex, n (%)	20 (40)	16 (32)	
Sudden symptom onset, n (%)	30 (60)	14 (28)	<0.05
Gradual symptom onset, n (%)	20 (40)	36 (72)	
Previous lumbar episodes, n (%)			0.659
None	7 (14)	10 (20)	
One episode	10 (20)	11 (22)	
Multiple episodes	33 (66)	29 (58)	

SD—standard deviation. *p*-values compare the two treatment cohorts. Treatment allocation was not randomized.

**Table 2:** SF-36 component scores and ODI values during six months of follow-up.

**Outcome**	**Group**	**Admission**	**3 Months**	**6 Months**	**Absolute Change, 0–6 Months**
SF-36 PCS	Non-surgical	35.5	44.7	50.8	+15.3
	Surgical	28.8	42.8	49.2	+20.4
SF-36 MCS	Non-surgical	40.6	44.8	52.6	+12.0
	Surgical	37.8	45.2	52.5	+14.7
ODI, %	Non-surgical	51.5	36.0	22.5	−29.0 pp
	Surgical	56.1	38.9	23.7	−32.4 pp

Higher PCS and MCS values indicate better health-related quality of life; lower ODI values indicate less disability. PCS—Physical Component Summary; MCS—Mental Component Summary; ODI—Oswestry Disability Index; pp—percentage points.

**Table 3:** Within-group change and between-group comparisons.

**Outcome**	**Within-Group RM ANOVA, Non-Surgical**	**Within-Group RM ANOVA, Surgical**	**Between-Group *p*, Admission**	**Between-Group *p*, 3 Months**	**Between-Group *p*, 6 Months**
SF-36 PCS	F = 450.221; *p* < 0.001	F = 490.721; *p* < 0.001	<0.001	<0.05	0.064
SF-36 MCS	F = 106.543; *p* < 0.001	F = 72.055; *p* < 0.001	0.104	0.871	0.323
ODI	F = 432.810; *p* < 0.001	F = 1341.180; *p* < 0.001	0.109	0.162	0.427

RM ANOVA assessed change across the three measurements within each group. Between-group comparisons were performed with the Mann–Whitney U test. No group-by-time interaction was reported in the source analysis.

## Data Availability

The data presented in this study are available on request from the corresponding author. The data are not publicly available because of privacy restrictions concerning patient information.

## Supplementary Materials

The following supporting information can be downloaded at https://www.mdpi.com/article/10.3390/jcm15218462/s1, Table S1: STROBE checklist for the cohort study; Table S2: SF-36v2 domain scores in the surgical and conservative treatment groups at rehabilitation admission and at 3-month and 6-month follow-up.

## Abbreviations

The following abbreviations are used in this manuscript:

SF-36	Short Form-36 Health Survey
PCS	Physical Component Summary
MCS	Mental Component Summary
ODI	Oswestry Disability Index
