# Supplementary material for: Comparative Outcomes of Surgical and Non-Surgical Treatment in Patients with Lumbar Radiculopathy: A Six-Month Analysis of Quality of Life and Functional Status
Source: J Clin Med. 2026 Oct 30;15(21):8462. doi: 10.3390/jcm15218462 (PMC13764604; doi:10.3390/jcm15218462)
Supplement: Supplementary file 1 [file jcm-15-08462-s001.zip › jcm-4576189-supplementary.pdf]

Supplementary Table S1. STROBE checklist for the cohort study

Comparative Outcomes of Surgical and Non-surgical Treatment in Patients with Lumbar Radiculopathy: A Six-Month Analysis of Quality of Life and Functional Status

Section	Item No.	Recommendation	Location in manuscript
Title and abstract	1	(a) Indicate the study's design with a commonly used term in the title or the abstract	Title/Abstract, p. 1
Title and abstract	1	(b) Provide in the abstract an informative and balanced summary of what was done and what was found	Abstract, p. 1
Introduction	Introduction	Introduction	
Background/rationale	2	Explain the scientific background and rationale for the investigation being reported	Introduction, pp. 2–3
Objectives	3	State specific objectives, including any prespecified hypotheses	Introduction, p. 3
Methods	Methods	Methods	
Study design	4	Present key elements of study design early in the paper	Methods, p. 3
Setting	5	Describe the setting, locations, and relevant dates, including periods of recruitment, exposure, follow-up, and data collection	Methods, pp. 3–4
Participants	6	(a) Cohort study—Give the eligibility criteria, and the sources and methods of selection of participants. Describe methods of follow-up<br>Case-control study—Give the eligibility criteria, and the sources and methods of case ascertainment and control selection. Give the rationale for the choice of cases and controls<br>Cross-sectional study—Give the eligibility criteria, and the sources and methods of selection of participants	Methods, pp. 3–4
Participants	6	(b) Cohort study—For matched studies, give matching criteria and number of exposed and unexposed<br>Case-control study—For matched studies, give matching criteria and the number of controls per case	Methods, p. 3; not a matched study
Variables	7	Clearly define all outcomes, exposures, predictors, potential confounders, and effect modifiers. Give diagnostic criteria, if applicable	Methods, pp. 3–4
Data sources/ measurement	8*	For each variable of interest, give sources of data and details of methods of assessment (measurement). Describe comparability of assessment methods if there is more than one group	Methods, pp. 4–5
Bias	9	Describe any efforts to address potential sources of bias	Methods, pp. 3–4; Discussion, pp. 9–10
Study size	10	Explain how the study size was arrived at	Methods, p. 5
Quantitative variables	11	Explain how quantitative variables were handled in the analyses. If applicable, describe which groupings were chosen and why	Statistical Analyses, p. 5
Statistical methods	12	(a) Describe all statistical methods, including those used to control for confounding	Statistical Analyses, p. 5
Statistical methods	12	(b) Describe any methods used to examine subgroups and interactions	Statistical Analyses, p. 5; treatment-by-time interaction not tested
Statistical methods	12	(c) Explain how missing data were addressed	Statistical Analyses, p. 5; no missing-data method reported
Statistical methods	12	(d) Cohort study—If applicable, explain how loss to follow-up was addressed<br>Case-control study—If applicable, explain how matching of cases and controls was addressed<br>Cross-sectional study—If applicable, describe analytical methods taking account of sampling strategy	Methods, p. 3; no loss to follow-up
Statistical methods	12	(e) Describe any sensitivity analyses	Statistical Analyses, p. 5; no sensitivity analysis reported

Section	Item No.	Recommendation	Location in manuscript
Results	Results	Results	
Participants	13*	(a) Report numbers of individuals at each stage of study—eg numbers potentially eligible, examined for eligibility, confirmed eligible, included in the study, completing follow-up, and analysed	Results, p. 6
Participants	13*	(b) Give reasons for non-participation at each stage	Methods, p. 3; all 100 enrolled participants completed follow-up
Participants	13*	(c) Consider use of a flow diagram	Results, p. 6; no flow diagram
Descriptive data	14*	(a) Give characteristics of study participants (eg demographic, clinical, social) and information on exposures and potential confounders	Results, p. 6; Table 1
Descriptive data	14*	(b) Indicate number of participants with missing data for each variable of interest	Results, p. 6; not explicitly reported; complete follow-up stated
Descriptive data	14*	(c) Cohort study—Summarise follow-up time (eg, average and total amount)	Results, p. 6; admission, 3- and 6-month assessments
Outcome data	15*	Cohort study—Report numbers of outcome events or summary measures over time	Results, pp. 6–7; Tables 2–3
Outcome data	15*	Case-control study—Report numbers in each exposure category, or summary measures of exposure	Results, pp. 6–7; Tables 2–3
Outcome data	15*	Cross-sectional study—Report numbers of outcome events or summary measures	Results, pp. 6–7; Tables 2–3
Main results	16	(a) Give unadjusted estimates and, if applicable, confounder-adjusted estimates and their precision (eg, 95% confidence interval). Make clear which confounders were adjusted for and why they were included	Results, pp. 6–8; unadjusted comparisons reported
Main results	16	(b) Report category boundaries when continuous variables were categorized	Not applicable
Main results	16	(c) If relevant, consider translating estimates of relative risk into absolute risk for a meaningful time period	Not applicable
Other analyses	17	Report other analyses done—eg analyses of subgroups and interactions, and sensitivity analyses	Results, pp. 6–8; no subgroup/sensitivity analyses reported
Discussion	Discussion	Discussion	
Key results	18	Summarise key results with reference to study objectives	Discussion, pp. 8–9
Limitations	19	Discuss limitations of the study, taking into account sources of potential bias or imprecision. Discuss both direction and magnitude of any potential bias	Discussion, pp. 9–10
Interpretation	20	Give a cautious overall interpretation of results considering objectives, limitations, multiplicity of analyses, results from similar studies, and other relevant evidence	Discussion, pp. 9–10
Generalisability	21	Discuss the generalisability (external validity) of the study results	Discussion, pp. 9–10
Other information	Other information	Other information	
Funding	22	Give the source of funding and the role of the funders for the present study and, if applicable, for the original study on which the present article is based	Funding, p. 10; no external funding

* Give information separately for cases and controls in case-control studies and, if applicable, for exposed and unexposed groups in cohort and cross-sectional studies.

Note: The checklist is completed against the current manuscript version. Where an item is not applicable, not reported, or was not performed, this is stated explicitly rather than inferred.

Supplementary Table S2. SF-36v2 domain scores in the surgical and conservative treatment groups at rehabilitation admission and at 3-month and 6-month follow-up.

SF-36v2 domain	Group	Admission	3 months	6 months
**Physical Functioning (PF)**	Non-surgical	42.2±19.98	66.4±14.71	82.3±10.06
	Surgical	26.5±10.16	62±11.02	80.3±6.73
**Role Physical (RP)**	Non-surgical	26.1±20.23	56.1±18.66	80.25±13.38
	Surgical	10.5±12.53	45±15.52	75.2±15.77
**Bodily Pain (BP)**	Non-surgical	28.1±15.37	52.4±13.99	74.1±15.45
	Surgical	11.88±9.87	53.8±17.69	71±16.66
**General Health (GH)**	Non-surgical	57.74±22.5	63.74±20.86	68.56±19.97
	Surgical	52.4±14.45	60.62±14.82	68.52±11.1
**Vitality (VT)**	Non-	40.38±18.57	51.1±19.55	72.7±22.25

	surgical			
	Surgical	28±17.78	54.7±18.19	64.9±21.65
**Social Functioning (SF)**	Non-surgical	44.5±17.71	67.5±17.13	85±16.37
	Surgical	26±18.36	60.7±16.94	81.5±13.66
**Role Emotional (RE)**	Non-surgical	50.7±32.16	70.5±24.47	85±15.97
	Surgical	39.8±25.38	68.5±13.6	89.3±12.38
**Mental Health (MH)**	Non-surgical	50.1±19.83	56.5±17.35	68.2±13.13
	Surgical	48±18.74	61.8±8.38	77.6±7.97

Note: Data are presented as mean values. Each treatment group included 50 participants. The eight SF-36v2 domain scores range from 0 to 100, with higher scores indicating better health-related quality of life. Assessments were performed at rehabilitation admission and after three and six months.
